# Elastohydrodynamic lubricant flow with nanoparticle tracking

**DOI:** 10.1039/c8ra09396b

**Published:** 2019-01-11

**Authors:** S. Jeffreys, L. di Mare, X. Liu, N. Morgan, J. S. S. Wong

**Affiliations:** Department of Mechanical Engineering, Imperial College London SW7 2AZ UK j.wong@imperial.ac.uk; Oxford Thermofluids Institute, Department of Engineering Science, St John's College, University of Oxford OX2 0ES UK; Shell Global Solutions UK Ltd Manchester M22 0RR Lancashire UK

## Abstract

Lubricants operating in elastohydrodynamic (EHD) contacts exhibit local variations in rheological properties when the contact pressure rises. Direct evidence of this behaviour has only been obtained by examining through-thickness velocity profiles *U*(*z*) of lubricants in a contact using luminescence-based imaging velocimetry. In the present study, nanoparticles (NPs) are added to polybutene (PB) as tracers to investigate the effect of pressure on the flow of PB in an EHD contact. By tracking NPs in the contact, particle velocity distributions *f*(*U*) under various pressures are obtained and found to be pressure dependent. Results show quantitatively that *f*(*U*) and *U*(*z*) are correlated and thus confirm that *U*(*z*) of PB changes from Couette flow to partial plug flow above a critical pressure. This confirmation highlights the complexity of lubricant rheology in a high pressure contact.

## Abstract 2

Lubricants operating in elastohydrodynamic (EHD) contacts exhibit local variations in rheological properties when the contact pressure rises.

## Graphical abstract

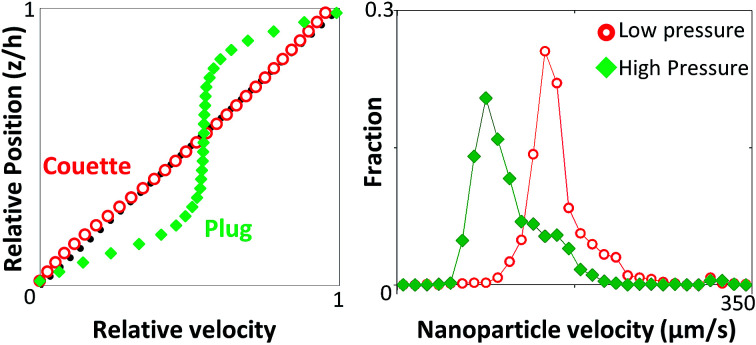

## Introduction

In many engineering components with non-conformal elements that both roll and slide together – *e.g.* gears, rolling element bearings and cam/follower systems – much of the friction loss originates in the elastohydrodynamic (EHD) lubrication regime.^1^ In EHD contacts a thin lubricant film (<μm) separates two surfaces in relative motion and is subject to a combination of high pressures (up to 3 GPa) and shear rates (up to 10^8^ s^−1^).^2^ Friction is a result of hydrodynamic losses as the lubricant film is sheared.

Under EHD conditions, even low molecular weight organic liquids can exhibit non-Newtonian behaviour.^3^ Current models used to interpret EHD friction are based on the assumption that, without thermal effects or until a critical shear rate is met, the density and viscosity of the lubricant are uniform across the film thickness.^1^ As a result, the lubricant velocity varies linearly through the thickness of the film as in a Couette flow. Under this assumption, the local shear rate is the same as the macroscopic velocity gradient inferred from the relative speed of the contact surfaces and the film thickness. Under the conditions experienced in EHD contacts, however, the shear can become localised *i.e.* regions of fluid that shear at different rates from the nominal value.^4–6^ An example is plug flow where the lubricant flows as a solidified plug at the mean entrainment speed, with the shear zones close to the walls.^7–10^

As a consequence of the uncertainty surrounding actual flow conditions, there is still disagreement as to which constitutive equation most accurately describes the rheology of lubricants in rolling-sliding contacts under EHD conditions.^1^ There is, therefore, a clear need for *in situ* experimental techniques which measure important physical quantities such as flow, pressure, temperature and viscosity in EHD contacts with good spatial and temporal resolution.^11,12^

The most successful velocimetry method in EHD has so far been from molecular tagging velocimetry (MTV). Reddyhoff *et al.*^13^ used fluorescence microscopy to study the lubricant flow in an EHD contact under pure rolling conditions. The average velocity of fluorophore-doped lubricant was monitored across the contact with a central film thickness of 200 nm. While this technique gives information on the average velocity through the contact, the technique is unable to distinguish the through-thickness velocity distribution.

**Fig. 1:**
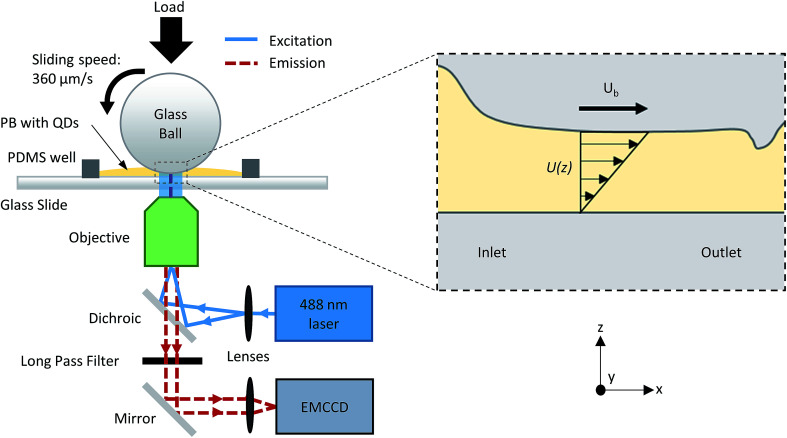
Schematic of nanoparticle imaging velocimetry setup. Experimental observation is conducted in the *x*–*y* plane. The black dashed line shows a section view along the contact centre of a classical EHD point contact. When load is applied, the glass ball elastically deforms to form a conformal contact region with the glass slide. Arrows represent a classical Couette shear velocity profile for pure sliding where *U*_b_ is the ball velocity.

The first through-thickness velocity profile measured in an EHD contact was by Ponjavic *et al.*^14^ using photobleached-fluorescence imaging velocimetry with fluorophore-doped lubricants. Briefly, a high energy, highly focused laser beam creates a tagged through-thickness column of lower intensity than the bulk lubricant. Convection of the fluid causes the tagged volume to change shape with time as shear is applied. Because of the configuration of EHD contacts, the spatiotemporal evolution of the column is viewed from the *x*–*y* plane (see Fig. 1). The velocity profile is then reconstructed from the intensity distributions through an optical flow reconstruction technique.^14^ Flow heterogeneity was observed in polybutene (PB). Subsequent investigations showed the effect of pressure^15^ and surface chemistry^16^ on PB in EHD contacts.

A similar luminescence-based velocimetry technique was later developed by capturing only phosphorescence signal from a lubricant doped with a phosphorescent probe.^17^ In this case, the tagged through-thickness column has high intensity compared to the rest of the lubricant. The enhanced temporal resolution allows for the flow of lower viscosity oils at higher entrainment speeds to be studied, benefitting from improved signal-to-noise ratio. The streamwise resolution of luminescence-based techniques is partly limited by the size of the laser beam used to bleach or excite the tagged volume. Furthermore, these techniques rely on the availability of suitable dyes.

The limitations stated above may partially be overcome by implementation of nanoparticle tracking, where the fluid is seeded with nanoparticles (NPs) whose motion are subsequently tracked as a function of time. NPs such as quantum dots have very high quantum yields, hence giving high signal to noise ratio and diffraction-limited resolution. The surface of the NPs can be functionalised to improve their solubility in the fluid of interest, making this method suitable for many fluid chemistry and for nanofluidic systems.^18^ Its versatility and high spatiotemporal resolution make single particle tracking an interesting proposition for visualising fluid flow. Microscale particle tracers have been used to study the rheology of lubricants. Bair *et al.*^4^ developed a rheometer capable of measuring the high pressure rheology of lubricants at similar pressures to those encountered in EHD lubrication. By tracking micro-sized dispersed particles (on the *x*–*z* plane, see Fig. 1 for coordinate axis), the authors observed shear localisation in a 150 μm gap at pressures up to 0.3 GPa. More recently, a PIV technique was developed to investigate particle entrapment in EHD contacts, showing the presence of backflows at the inlet^19^ (see Fig. 1 for location of the inlet). Micro-sized graphite particles have also been tracked using interferometry, although the number of particles tracked was too small to make a conclusive observation of the lubricant flow.^9^

NPs with diameter of few nm are attractive tracers for flow studies in EHD, because of their small size compared to both film thickness for most lubricants in engineering conditions and the size of the laser beams used in fluorescence spectroscopy. The use of QDs has been proposed for *in situ* measurements of temperature and pressure.^20^ The photoluminescence sensitivity of QDs in highly confined liquids was investigated. However NPs have not been used to investigate lubricant flow in a contact *in situ*. This paper demonstrates the use of NPs for flow investigation of a model lubricant in EHD contacts. The results are compared to through-thickness velocity profiles obtained by photobleached-fluorescence imaging velocimetry to validate the effect of pressure on the flow of the model lubricant.

## Experimental

### Materials

The lubricant used in this study is polybutene PB2300 (Sigma-Aldrich). PB2300 is a highly viscous oligomer and consists largely of repeating monomers of isobutylene (∼90%), together with monomers of 1-butene and 2-butene. It has a number average molecular weight of 2300 g mol^−1^. The model lubricant has a pressure–viscosity coefficient of around 32 GPa^−1^, measured from film thickness measurements using interferometry.^21^ The flow behaviour of PB2300 is pressure-sensitive and a transition from Couette flow to partial plug flow takes place as the normal pressure is increased.^15^

Alkyl functionalised, hydrophobic CdSeS/ZnS alloyed QDs of diameter 6 nm with fluorescence emission peak at 540 nm (Sigma-Aldrich 753777) were used as NP tracers. Results from dynamic light scattering using 0.1 mg ml^−1^ in toluene (QD stock solution) showed a size distribution peak at 6 nm. It is important to prevent perturbations to the flow behaviour, and in this case individual NPs are considered small when compared with the film thickness (∼250 nm).

The testing sample is prepared by mixing QD stock solution to PB2300. The QD stock solution is first diluted with toluene to ensure a small concentration of NPs in the final sample. The diluted stock solution is added to PB to create a mixture consisting of 1.73 g toluene, 5 × 10^−8^ mg QDs and 25.4 g of PB2300. The mixture is stirred for 120 hours at 100 °C. Toluene removal from the mixture is attempted by heating the mixture at 150 °C, followed by vacuum pumping for 30 minutes. The toluene removal process is repeated at least 3 times, until no bubble is observed during the vacuum pumping step. Test solutions containing NPs are referred to as NP solutions. A control solution without QDs is also prepared using the same method as the NP solutions, including the mixing and toluene removal processes. It is assumed that the amounts of trace toluene in the NP solutions and the control solution are the same. The toluene removal process does not remove all the toluene from the solution as the viscosities of the NP and control solutions (∼118 Pa s at 25 °C) are lower than the viscosity of PB2300 (480 Pa s at 25 °C). By applying the Refutas equation,^22^ the residual toluene in the NP solutions is estimated to be around 3 wt%. Based on this estimate, the NP solution has 1.9 × 10^−10^ wt% of NPs. Taking the density of CdSe and the diameter of NPs as 5.816 g cm^−3^ and 6 nm respectively, the concentration is estimated to be 2.8 × 10^6^ particles per ml. Another solution with a concentration of 4 × 10^12^ particles per ml is prepared. These NP solutions are referred as low concentration (LC) and high concentration (HC) respectively. It should be noted that both concentrations are low to ensure the rheology of the lubricant is not affected. Using low concentrations will also minimise NP aggregation.

### NP tracking in an EHD contact

**Fig. 2:**
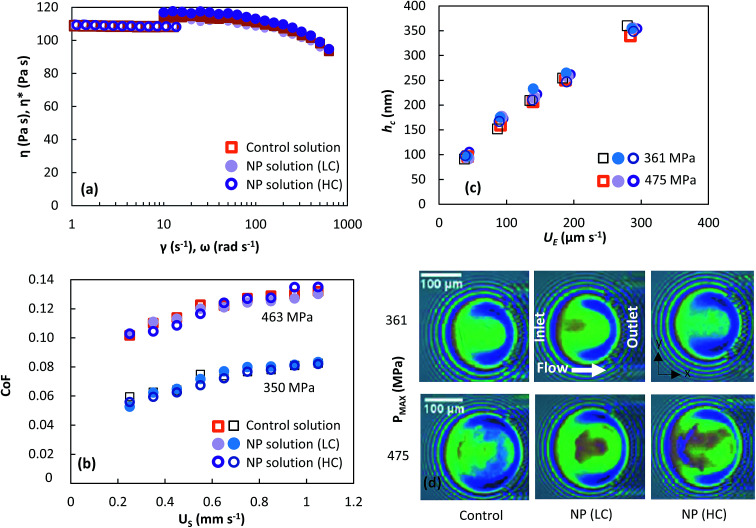
Effect of NPs on lubricant properties. (a) Steady-state and oscillatory rheological data for NP and control solutions. Shear viscosity (*η*) *versus* shear rate (*γ̇*) (open symbols); and complex viscosity (η*) versus rotational frequency (*ω*) (closed symbols). (b) Coefficient of Friction (CoF) *versus* disc sliding speed (*U*_S_). (c) Central film thickness (*h*_c_) *versus* entrainment speed (*U*_E_) (d) SLIM images at an entrainment speed (*U*_E_) of 133 μm s^−1^. Flow is in the *x*-direction.

A pure sliding EHD contact is created by loading a rotating 3/4′′ borosilicate glass sphere (PCS Instruments, arithmetic mean roughness (*R*_a_) = 5 nm) against a stationary glass slide (VWR, *R*_a_ = 0.5 nm). A PDMS well surrounds the contact and is filled with the lubricant solutions to ensure operation in fully flooded conditions. The glass sphere rotates at a fixed sliding velocity of 360 μm s^−1^ such that the entrained lubricant fully separates the two surfaces. A normal load, *W*, ranging between 8 and 35 N is applied. The shape of the resulting contact is circular and has been verified by interferometry (see Fig. 2d). The pressure *P* experienced by the fluid is estimated by Hertzian contact mechanics. This is parabolic and depends on position:1
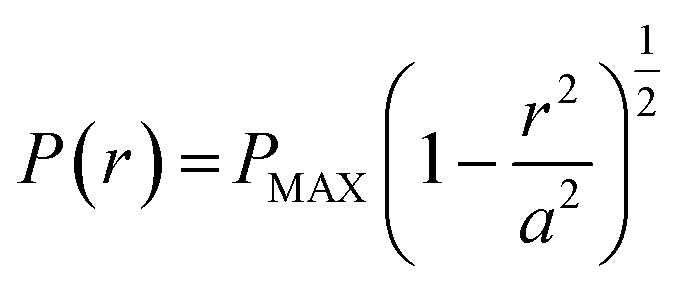
where *r* is the distance from the centre of the contact and *a* is the contact radius. *P*_MAX_ is the peak pressure and is equal to:2
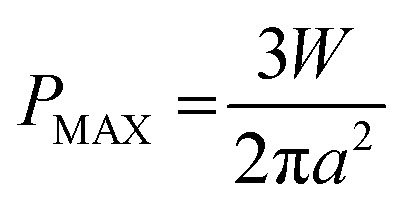


Taking the Young's modulus (*E* = 70 GPa) and Poisson's ratio (*ν* = 0.2) for glass, the applied normal loads correspond to a Hertzian peak pressure range between 283 MPa and 463 MPa. The Hertzian contact radius *a* is estimated between 116 and 190 μm respectively. All measurements are performed at room temperature (25 ± 1 °C). Heat generation due to shear has previously been estimated and shown to be negligible due to the low velocities employed.^14^ The viscosity of lubricants has been shown to increase locally with pressure in EHD conditions (known as the piezo-viscous effect) using fluorescence-based techniques.^23–26^ The diffusion of NPs is estimated to be negligible due to the high viscosities of our test solutions. Based on previous investigations, the fluid may experience shear thinning, particularly at high pressures.^15^

A schematic of the experimental setup is shown in Fig. 1. The tribological contact is imaged using a Zeiss Axiovert 200 M inverted microscope with a 10× (0.25 NA) or 20× (0.4 NA) objective. Cyan (488 nm at 12 mW on stage) solid state laser, operating in TEM00 mode, is used to excite the NPs. An Andor iXon3 860 electron multiplying charged coupled device (EMCCD) camera collects the fluorescence emission from the NPs. For the 20× objective, the depth of field is 2.96 μm, while the thickness of the lubricant film in the EHD contact for the experimental conditions used in this work is less than 1 μm. With sufficient signal-to-noise ratio, all NPs in the EHD film should be observed clearly. Images are acquired using an exposure time and cycle time of 10 and 12 ms respectively. All images are background corrected to remove any fluorescence signal from the glass substrates. NPs are identified and tracked using the software ImageJ.^27^ Custom FORTRAN code is used to generate the trajectory and the velocity of the NPs. Only NPs which can be tracked for at least 20 consecutive frames are included in the analysis, however the results are not affected by the track length. Experiments were repeated three times for each test condition. Results are reproducible.

### Characterisation of EHD films

To successfully use NPs as fluid tracers, it is important they are kept at low concentration such that they do not alter the rheology of the lubricant. The viscosity of PB is obtained with a Discovery Hybrid Rheometer (TA instruments). The setup uses two 25 mm parallel UHP steel plate with 800 μm geometry gap. The top plate rotates as the bottom plate remains stationary. First, a flow test is completed to measure the shear viscosity, as the shear rate *γ̇* is increased from 1–80 s^−1^. An oscillatory test is then completed to measure the complex viscosity. Here the top plate oscillates at a controlled frequency from 1–100 Hz. The shear-dependence of the viscosity is obtained by applying the Cox–Merz rule^28^ to the oscillatory data: *η*(*γ̇*) = η*(*ω*) when *γ̇* = *ω*, *ω* is the rotation frequency in rad s^−1^. All tests are performed at 25 °C.

A tribometer (CETR-UMT2) is used to measure friction. In contrast to the experimental setup described in the previous section, for friction measurements a glass disc rotates while a glass sphere is fixed to a force sensor. The effect of the moving surface on friction measurements is found to be minimal. A normal load ranging between 5 to 35 N is applied, corresponding to a Hertzian peak pressure ranging from 242 to 463 MPa. At each load, the sliding speed varies from 250 to 1050 μm s^−1^.

Lubricant film thickness in an EHD contact is obtained using both optical interferometry and spacer layer imaging method (SLIM).^29^ The former provides the central film thickness and the latter the geometry of the lubricant film. Both methods are based on the principle of multiple beam interferometry^30^ and are implemented with an EHD2 ultrathin film measurement system (PCS-instruments). In order to apply interferometry, reflective surfaces are necessary. Tests are conducted with a 3/4′′ diameter steel ball and a glass disc coated with a semi-reflecting chromium layer, as the contact is illuminated using a white light source.^29^ The refractive index of the lubricant is set at 1.5. Lubricant film thickness is measured under pure sliding conditions at room temperature. Here the disc rotates and the ball remains stationary. The entrainment speed, which is the mean speed of the surfaces given as half the sliding speed, ranged between 40 and 300 μm s^−1^. The normal load applied ranged between 5–16 N, corresponding to peak contact pressures of 322 and 475 MPa using the following material properties for steel (*E* = 220 GPa, *ν* = 0.3).

Photobleached-fluorescence imaging velocimetry was conducted on the control solution to determine the through-thickness velocity distribution, *U*(*z*), at the contact centre.^14^ Nile red (Sigma-Aldrich) was dissolved into the control solution at 1 mM by magnetic stirring for 5 hours at 150 °C. The fluorophore-doped control solution is entrained into an EHD contact, and observed using an inverted microscope. A Spectra-Physics Cyan 488 nm is used to create a tagged column by photobleaching and a Spectra-Physics Excelsior 532 nm (15 mW on stage) is used to observe the spatial-evolution of the tagged column. Synchronous averaging is applied to improve the signal-to-noise ratio of images. Image sequences consisting of 20 images were acquired using a photobleaching, exposure and cycle time of 6, 1, and 5 ms respectively. All other conditions match precisely with the NP tracking experiments. Experimental intensity distributions are then compared against those generated by a numerical algorithm.^14^ The intensity profile of the tagged column at time = 0 is assumed to be Gaussian. The algorithm estimates *U*(*z*) as the fluid is modelled as a stack of infinitesimal fluid layers which flow parallel to the contact surfaces, where *z* is the through-film distance between the position of a fluid layer and the stationary glass slide. An iteration process minimises the difference between experimental and simulated intensity distributions to produce a final reconstructed through-film flow profile.^14^

## Results and discussion

### Effect of NPs on lubricant properties

The bulk rheological properties of the NP and control solutions are shown in Fig. 2a. Discontinuity occurs due to a change in test modes, giving rise to slightly different viscosities. Each solution exhibits Newtonian behaviour at low shear rates displaying constant shear viscosity. At high shear rates above 50 s^−1^, all solutions show shear thinning behaviour. The NPs have no effect on the viscosity. Lubricant viscosity governs friction and the film thickness across the contact. The coefficient of friction is affected more by an increase in pressure or sliding speed than the addition of NPs, as seen in Fig. 2b. The central film thickness values are similar for both the NP and control solutions (see Fig. 2c). The corresponding contact sizes observed using SLIM (see Fig. 2d) also agree with Hertzian contact mechanics for an EHD point contact given by:3
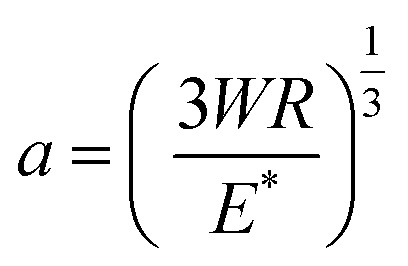
where *R* is the effective radius (4.8 mm) and E* is the combined elastic modulus (72.9 GPa). All pressures tested give the same conclusion. In Fig. 2c, central film thickness measurements overlap for both the control and NP solutions for all entrainment speeds. For Fig. 2d, at low pressure (Fig. 2d top row), the film thickness is constant across the central region of the contact. At high pressure (see Fig. 2d, bottom row) the central film thickness region is still rather uniform. However the film thickness becomes non-uniform particularly near the inlet as seen by the small crescent-shaped dimples that represent larger film thickness values. Dimples at the inlet have previously been reported to be linked to the non-Newtonian rheology.^7,31^ At all pressures the NP solutions give similar film shapes as the control solution. At the same test condition, similar film thicknesses suggest similar local average flow velocities.

One of the biggest challenges when adding NPs to the lubricant is maintaining the stability of the dispersion. No aggregate is observed during SLIM measurements under white light. Since the thickness of the lubricant film in the particle tracking experiments is small (∼250 nm), a build-up of aggregates may occur near the inlet. If this was the case the interference pattern in Fig. 2d would be disrupted; this is not observed. While these observations do not rule out the possibility of NP aggregates in the lubricant, they indicate that any aggregates must be much smaller than the wavelength of light. Furthermore larger aggregates, if they exist, may flow around rather than being entrained into the contact. Aggregation and NP flow are discussed in the following sections of the report.

There is no clear difference in rheology, friction and lubricant film thickness between the NP and control solutions and therefore it is assumed the NPs do not affect the lubricant flow. This is an advantage of working with low NP concentrations.

### Particle density and aggregation

While NPs are nominally 6 nm in diameter, they appear much larger due to the diffraction-limited resolution of the applied fluorescence imaging method. Particle tracking experiments are conducted using the HC NP solution (4 × 10^12^ particles per ml), since the dilute solution resulted in too few particles entering the contact to make conclusive observations.

The dispersion stability of NPs in colloidal suspensions can be affected when physical processes bring NP surfaces into contact with each other to form aggregates.^32^ NP surfaces are often functionalised to stabilize NPs against aggregation, however controlling a uniform NP dispersion is difficult and functionalisation may not always be very effective.^33,34^ It is worth mentioning functionalisation may promote aggregation in some cases.^35^

**Fig. 3:**
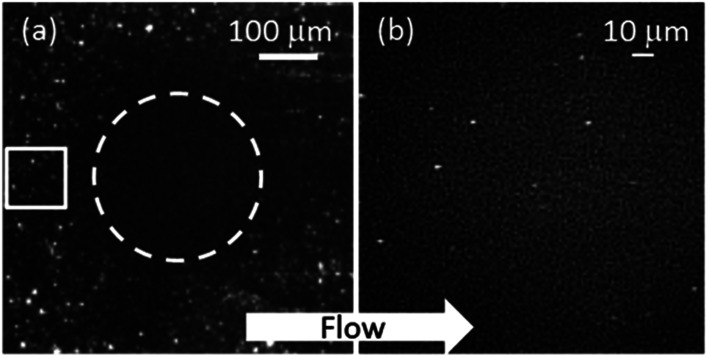
Single image in nPIV sequence (a) particle identification at inlet (solid line represents region of interest (ROI = 100 × 100 μm^2^); (b) NPs imaged at contact centre (within dashed line in (a)).

To assess the degree of NP aggregation, the NP size in the HC solution is estimated. Consider the region of interest at the inlet of an EHD contact as enclosed by the white square in Fig. 3a. There, the film thickness is about 2 μm and should contain roughly 8 × 10^4^ particles. If each white spot in Fig. 3a represents a particle, however, only 30 particles are identified inside the square using the ImageJ particle identification algorithm.^27^ This could be due to NP aggregation, with larger aggregates appearing brighter. In this case, individual particles and small aggregates might not be detected and the aggregate number density is underestimated. Using assumptions: (1) concentration of NPs at the inlet is the same as the rest of the lubricant; (2) each particle identified is an aggregate and (3) all aggregates are spherical and of the same size, then each aggregate would be made of ∼2.6 × 10^3^ NPs. Assuming dense packing of equal spheres, each aggregate has an estimated average diameter of 92 nm. This is an upper bound of average aggregate size, as the aggregate number density is dependent on the threshold set for particle identification. Also if multiple aggregates are close to each other (within the diffraction limit), they will be counted as one.

Particle identification is carried out in the same way at the centre of the contact (see Fig. 3b). There are few particles in the contact since the volume of the lubricant in the contact is very small. The intensities of particles inside the contact are low in comparison to the emission signal captured outside the contact. This may suggest that particles are smaller in the contact than aggregates outside. Since all particles in the contact lie on the focal plane, variations in intensities are due to differences in aggregate size.

### Monitoring flow profile with photobleached-fluorescence imaging velocimetry

**Fig. 4:**
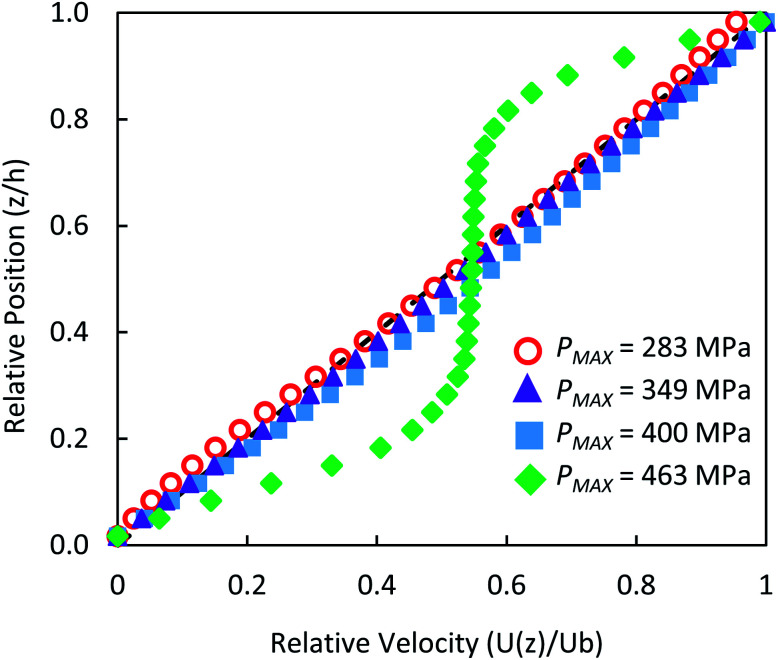
Measured through-thickness velocity profiles *U*(*z*) obtained for control solution using photobleached-fluorescence imaging velocimetry. The dash line corresponds to the linear Couette flow profile.

The through-thickness velocity profiles obtained for the control solution (see Fig. 4) are pressure dependent. At low contact pressures (<400 MPa), the velocity distribution resembles Couette (linear) flow. The velocity profile displays a constant shear rate and obeys the no-slip boundary condition. The shape of the velocity profile changes drastically in the high pressure case, showing partial plug flow behaviour. Here the local normalised shear rate is no longer constant through the thickness of the film. The flow profile is symmetric around the centre of the film 
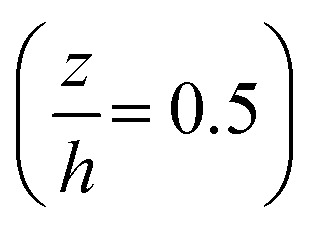
, where the fluid adjacent to the walls experiences high shear rates. However at the centre of the film between 
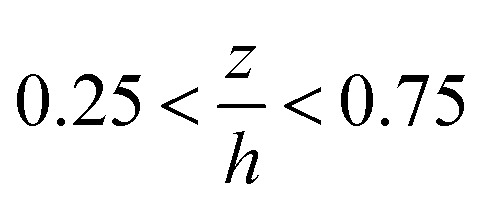
, the shear rate is approximately constant and is very small. This is similar to the partial plug flow profile previously presented at the same conditions,^15^ showing the addition of toluene in PB has no effect.

### NPs for flow velocimetry

For NPs to be used as tracers, the displacement of NP should reflect the fluid flow. To check the validity of this assumption, the Stokes number Sk is calculated. This is given as:4
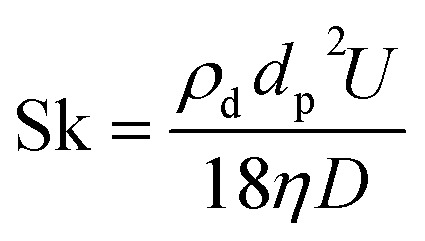
where *ρ*_d_ is the particle density, *d*_p_ is the particle diameter, *U* is the fluid velocity, *η* is the dynamic viscosity and *D* is the characteristic dimension of the flow (*h*_c_ = 250 nm). Assuming the particle diameter to equal the thickness of the lubricant film (*d*_p_ = 250 nm) and there is no piezoviscous effect (*η* = 118 Pa s), using the entrainment speed *U* = 180 μm s^−1^ gives a Sk = 1.2 × 10^−10^. The Stokes number of the NPs is in fact much smaller than this value because the diameter of even the largest aggregate is much smaller than *D*. Since Sk ≪ 1, the particles follow the streamlines.

Before proceeding further, it is worth describing what our expected observations were. Assuming a uniform particle density distribution and the lubricant obeys Couette flow with no slip boundary condition (see low pressure case in Fig. 4), particle velocities must range from 0 (stationary slide) to 360 μm s^−1^ (ball velocity, *U*_b_), with the number of observed particles increasing linearly with particle velocity over a fixed period of time. Any deviation from such relationship would suggest a different flow behaviour. For example: for plug flow, all particles would travel at the same velocity. This would result in a velocity distribution graph consisting of a vertical line at the entrainment speed (*U*_E_).

**Fig. 5:**
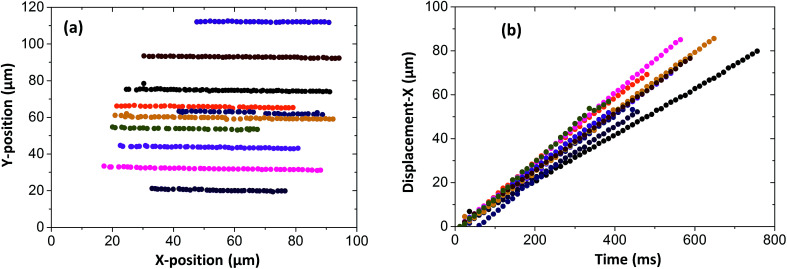
Individual tracking in elastohydrodynamic contact for 10 NPs (a) NPs tracked at 100 × 100 μm^2^ region of interest at contact centre. NPs travel from left to right; (b) displacement in flow direction (*x*) *versus* time. Colours used in (a) correspond with (b).

**Fig. 6:**
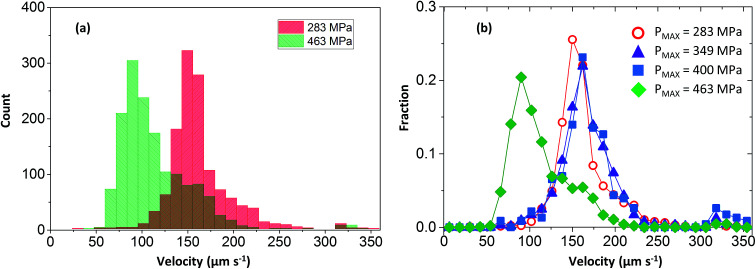
NP velocity distributions and count fractions for nanoparticle tracking experiments (a) NP count *versus* velocity for low and high pressure condition; (b) NP count fraction *versus* velocity.

Particles were tracked in a 100 × 100 μm^2^ region of interest at the contact centre (see Fig. 5a), where the normal pressure experienced by the fluid is close to the maximum pressure in the contact (see eqn. (1)). Particle velocities were obtained by plotting their displacement *versus* time (see Fig. 5b). The NPs travel at a range of speeds, as shown by the different slopes of the displacement–time curves in Fig. 5b. The velocity histogram for tracked NPs is shown in Fig. 6a. For clarity, only velocities at high and low pressures are shown. Distributions are then normalised with the total number of tracks captured at each respective pressure (see Fig. 6b).

Three important observations are recorded. Firstly all velocity distributions are non-linear. Secondly, bell-shaped velocity distributions centred around the entrainment speed are obtained in conditions where the peak pressure is less than 400 MPa. Thirdly, at high pressure where the fluid exhibits partial plug flow, the velocity distribution becomes biased towards lower velocities.

We have demonstrated that NPs do not perturb the flow behaviour of PB (see Fig. 2). Through-thickness flow profile shows the flow is Couette at peak pressures below 400 MPa (see Fig. 4) even though the NP velocity distributions are non-linear (see Fig. 6). Hence either the assumption that NP flow is governed only by viscous forces or that NPs are homogenously distributed is invalid. Although small, the Reynolds number can never be exactly zero and inertial effects on particle dynamics must be considered. The characteristic time scale to establish a non-uniform concentration profile due to inertial migration in our system would however be much larger than our experimental time frame. Therefore lateral migration is dismissed.^36–39^

To describe the recorded observations, it is possible to derive a set of formal relations between particle velocity distributions *f*(*U*), particle concentration profiles *C*(*z*) and fluid flow velocity distributions *U*(*z*). In an interval of time d*t*, the number of particles d*N* crossing a given station is defined as:5
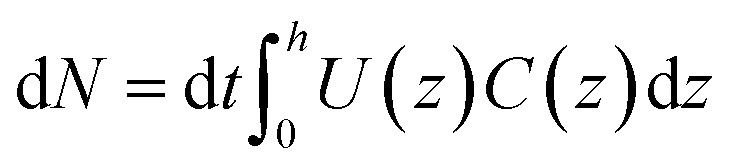
where *U* is the particle velocity and *C* is local concentration, both functions of the film thickness (*z*). The particle count rate, *Ṅ*, is then:6
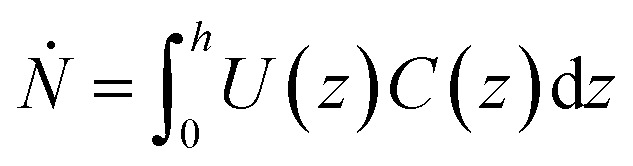


If the fluid velocity profile is monotonic then:7
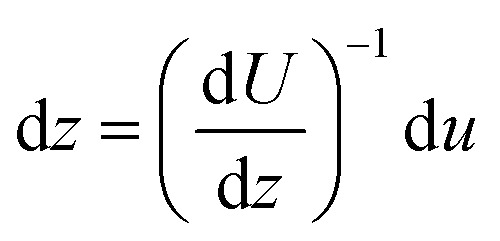
8
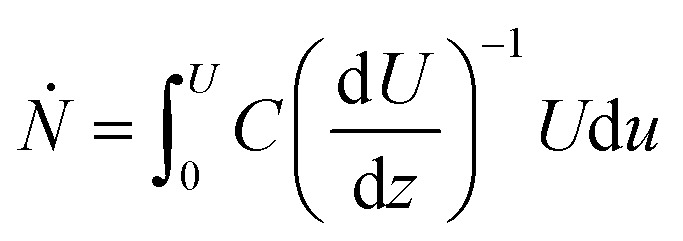


The fraction of particle counts (*f*(*U*)) with velocity in the interval [*U*,  *U* + d*u*] is therefore:9
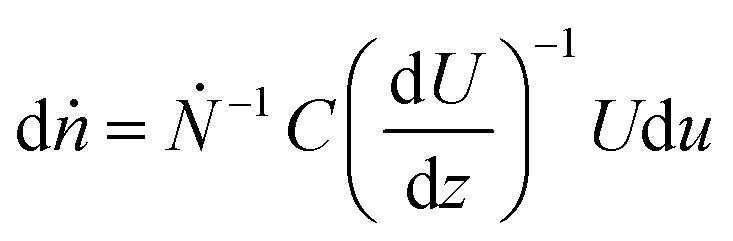
where d*ṅ* is the particle count rate fraction. For Couette flow:

10 d*ṅ* = *f*d*u* = (*γ̇Ṅ*)^−1^*CU*d*u*

**Fig. 7:**
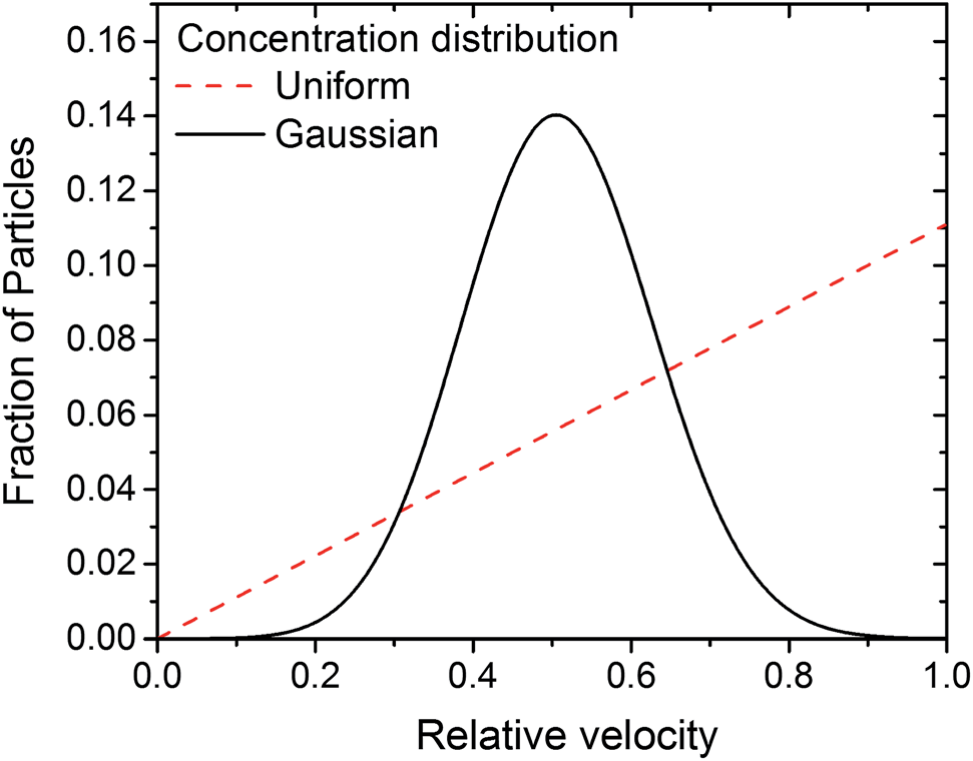
Expected particle velocity distribution *f*(*U*) based on eqn (10), applying either a uniform (dash line) or Gaussian (solid line) initial particle concentration distribution, assuming the flow is governed by Couette shear.

Based on eqn (10), if particles are uniformly distributed across the thickness of the lubricant film; *i.e. C* is constant, *f* should have the same shape as *U* as *f* ∝ *U*. This is demonstrated in Fig. 7. If the fluid obeys Couette flow, a uniform *C*(*z*) gives rise to a *f*(*U*) that is linear (dash line). If, however, *C*(*z*) is Gaussian with maximum at *z*/*h* = 1/2, *f*(*U*) is Gaussian skewed towards slightly higher velocity (solid line). The experimental evidence for Couette flow (see Fig. 6b) shows *f*(*U*) is in fact a bell shaped distribution suggesting *C* may not be uniform.

Assuming no change in total counts, if we now examine two stations where the velocity profile has been modified but there is no change in the mean shear rate, the ratio between count fractions in the interval [*U*, *U* + d*u*] is given as:11
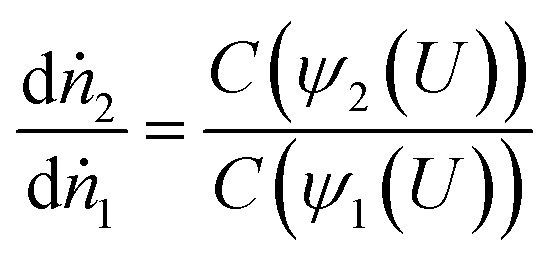
where *ψ*_1_ and *ψ*_2_ are the streamfunctions at station 1 and 2 respectively. Based on this formalisation above, one may predict particle velocity distribution at station 2 if information at station 1 is known. To apply this to our test geometry, station 1 is taken as a position in the contact near the inlet, where pressure is low and Couette flow applies.^40^ To simplify the analysis, the results presented below assume *C*(*z*) is Gaussian with mean *μ* at 
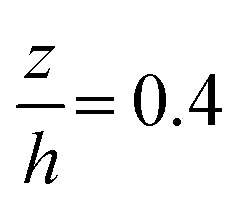
 and standard deviation *σ* = 0.35. Station 2 is at the centre of the contact. Predictions on particle velocity distributions at Station 2 are made based on fluid flow profiles in Fig. 4.

**Fig. 8:**
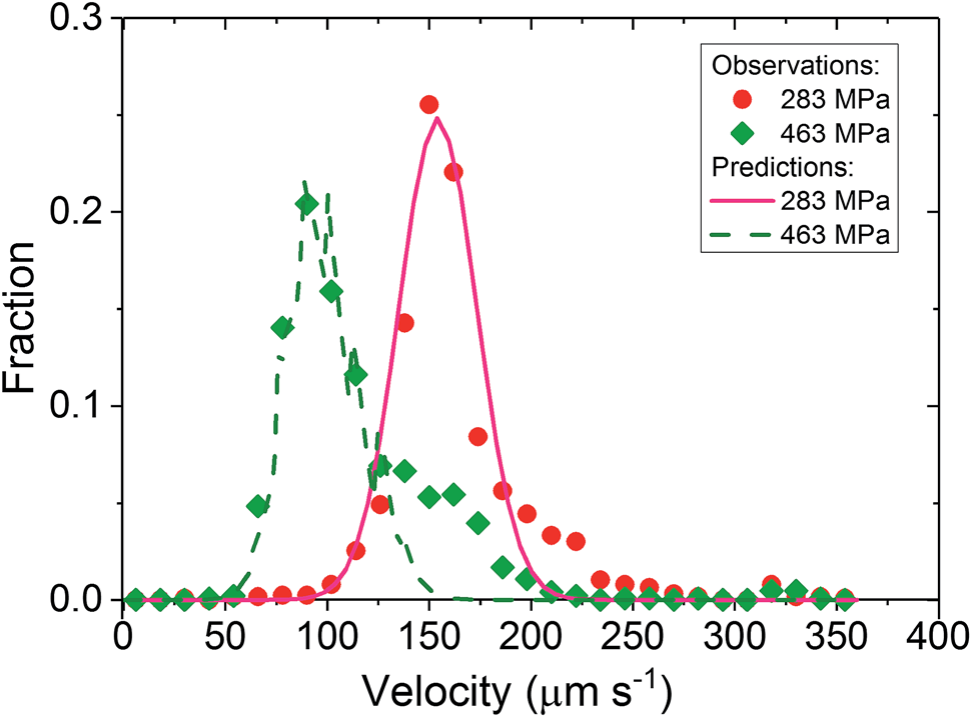
Predicted *f*(*U*) (lines) and experimental *f*(*U*) (markers) at Station 2. Predicted *f*(*U*) are based on eqn (11) using the simplified assumption of a Gaussian concentration distribution *C*(*z*) at Station 1 (inlet).

At *P* = 283 MPa, PB at station 2 follows Couette flow. Despite the simplified assumptions, the predicted *f*(*U*) (solid line, Fig. 8) and experimental *f*(*U*) (circles, Fig. 8) match reasonably well as the choice of *C*(*z*) is based on experimental results, both *U* and *f* obtained in at this pressure. Interestingly with the same *C*(*z*), the predicted (dash line, Fig. 8) and experimental (diamonds, Fig. 8) *f*(*U*) match at *P* = 463 MPa where PB exhibits partial plug flow. This shows that the difference in particle velocity distributions at different *P* is driven by a change in the fluid flow profile. These results confirm our results from photobleached-fluorescence imaging velocimetry that the flow of PB transits from Couette flow to partial plug flow as pressure increases. While the predicted and experimental *f*(*U*) in Fig. 8 are comparable, the detailed shape of *f*(*U*) would depend on the actual particle concentration distribution at the inlet of the contact. It is currently unclear why *C*(*z*) is not constant. The interaction between the wall and the particle may play a role. In this study, particles were rarely seen on the surface. Adhered particles were only found at surface defects. These were identified easily and were removed from the analysis. This suggests that the interaction between the surfaces and the NPs are likely to be repulsive. Since both rubbing surfaces are glass, one would then expect a depletion of NP aggregates to the same extent, with uniform through-thickness particle distribution in the rest of the fluid, which is not the case in this work. While NP-surfaces interactions may not be the determining factor, it may nevertheless contribute to the uneven particle distributions in the *z*-direction.

NP aggregation may also contribute to non-uniform particle concentration distribution. During the particle tracking process, we are tracking the centre of mass of these NP aggregates. If they are of relatively large sizes, their velocities will represent velocity of the fluid away from the wall, giving the impression that the particle concentration away from the wall is higher. This effect may also be compounded by the fact that aggregate size might not be uniform. Alternatively, the flow field at the inlet may influence the through thickness locations of those NP aggregates that can enter the contact.^41^ It has been shown using micron sized particles that under pure rolling conditions the position of a particle at the inlet governs whether it will be entrained into the contact or it will be swept to the side of the contact.^42^ A similar effect may also apply in our study. While there is uncertainty in the *C*(*z*) at the inlet and its origin, the conclusion of the observed particles velocity distribution at different pressures is a consequence of a change of fluid flow behaviour remains. This confirms a transition from Couette to partial plug flow when pressure increases.

**Fig. 9:**
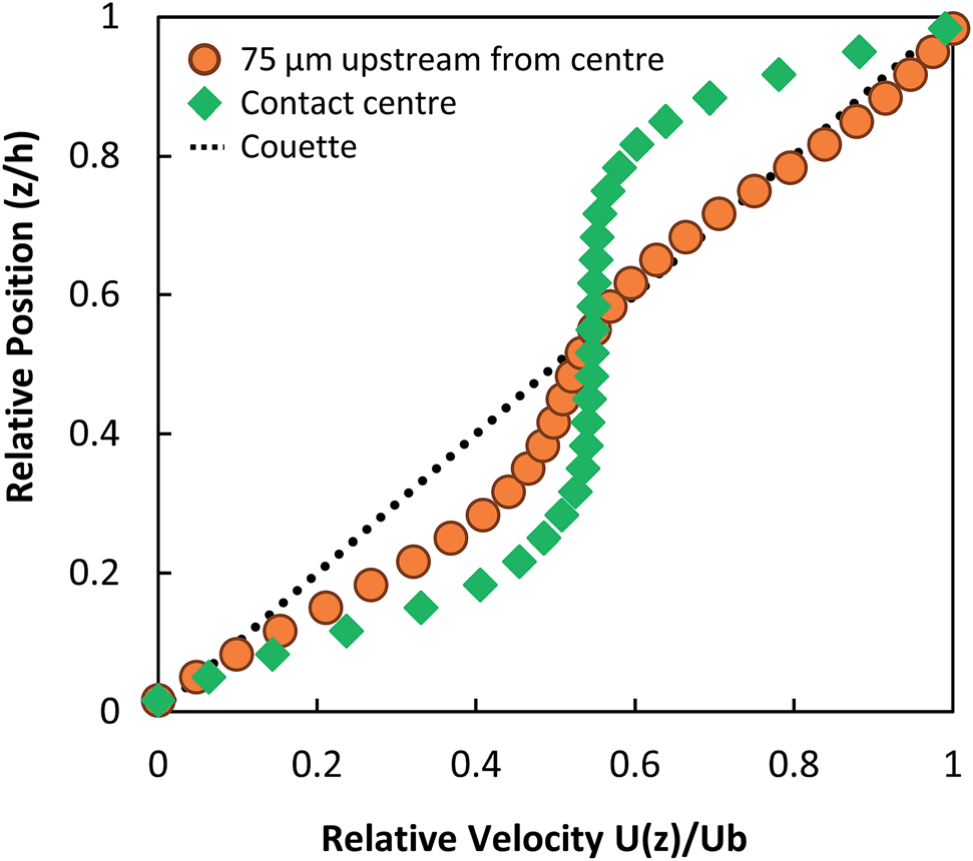
Measured flow profiles using photobleached-fluorescence image velocimetry at different positions within the contact at high pressure (*P*_MAX_ = 463 MPa). Local flow profiles at the contact centre (green diamond) and at a radial position of *r* = 75 μm (orange circles) from the contact centre (upstream towards the inlet) are shown.

Local pressure in the contact changes according to eqn (1). As a result, local flow profile changes in the contact.^40^ Hence particles trajectories reflect the change of flow profile across the contact due to the local pressure change. This poses the question of whether the fluid flow profile experienced by the tracked NPs at the centre of the contact (the view shown in Fig. 6b) has changed significantly. Fig. 9 compares flow profiles taken at the centre of the contact (*P* = 463 MPa) and a position 75 μm upstream (*P* = 425 MPa). The upstream location was chosen to examine fluid flow just outside of the view in Fig. 6b. At this upstream location, slight derivation from the Couette flow profile is observed (circles, Fig. 9). Since the pressure is higher nearer the centre where NP tracking is conducted, the lubricant flow will remain a partial plug flow. Note, the local pressure variation is the smallest at the centre of contact, especially when the *P*_MAX_ is high. This means in the region where results in Fig. 5, 6 and 8 are obtained, the flow profiles and the particle flow can be linked to pressure most directly. The validity of this approach is supported by (1) results in Fig. 5b where velocities of each tracked particles are constant and (2) particles velocity distributions are similar at low pressure (*P* ≤ 400 MPa, see Fig. 6b).

## Conclusions

In this work, lubricant flow is observed *in situ* with hydrophobic QDs as tracers in an EHD contact. Despite aggregation, NPs were entrained and tracked successfully to determine the effect of pressure on the flow characteristic of polybutene (PB).

Previous work using photobleached fluorescence imaging velocimetry, which is reproduced in this work, shows that polybutene exhibits Couette and partial plug flow at low and high pressure respectively. Using NP tracking offers an alternative to validate these results.

NP velocity distributions *f*(*U*) in PB are pressure dependent. A substantial shift in *f*(*U*) is observed when the pressure is sufficient high. A protocol has been developed which links through-thickness flow profile *U*(*z*) obtained using FPIV with *f*(*U*). Our results shows quantitatively that the shift in *f*(*U*) is consistent with the observed change in *U*(*z*) from Couette to partial plug flow when pressure reaches 463 MPa. This offers further support that lubricants experiencing high pressure may exhibit local flow heterogeneity. This possibility has not been given much consideration for most rheological models, and their origins and consequences must be investigated further.

The details of the particle velocity distribution *f*(*U*) from NP tracking is governed by the particle concentration distribution at the inlet, which in part is affected by the particle size distribution and the flow field at the inlet. Hence *f*(*U*) alone cannot be used to identify through-thickness flow profile unless *C*(*z*) in the inlet is known. It is, however, possible to estimate *C*(*z*) in the inlet if *f*(*U*) is obtained from a known through-thickness flow profile, such as Couette flow. In this case, *f*(*U*) can provide valuable information of local fluid flow.

## Conflicts of interest

There are no conflicts to declare.

## Supplementary Material
